# The Effect of Plaque Detectors on the Color Stability of Two Types of Restorative Materials

**DOI:** 10.1111/jerd.13420

**Published:** 2025-01-25

**Authors:** Claudia Mazzitelli, Gaetano Paolone, Uros Josic, Edoardo Mancuso, Alessandro Vichi, Ginevra Pastremoli, Annalisa Mazzoni, Lorenzo Breschi, Tatjana Maravic

**Affiliations:** ^1^ Department of Biomedical and Neuromotor Science (DIBINEM), Alma Mater Studiorum University of Bologna Bologna Italy; ^2^ Department of Dentistry, IRCCS San Raffaele Hospital and Dental School Vita Salute University Milan Italy; ^3^ Oral Biomaterials, Dental Academy University of Portsmouth Portsmouth UK

**Keywords:** color stability, colorimeter, glass‐ionomer cement, plaque detector, resin composite

## Abstract

**Objective:**

To investigate the color stability of a one‐shade resin‐based composite material (RC) and a glass‐ionomer cement (GIC) after staining with plaque detectors (PDs) with different formulations and delivery forms.

**Materials and Methods:**

Rectangular‐shaped specimens (7 × 3 × 2 mm) were produced with RC (Venus Diamond One, Kulzer) and GIC (Fujy IX GP, GC) (*n* = 30). Further, the following PDs were used on the specimens: (1) tablets (T; Plaq‐Search, TePe); (2) mouthwash (M; Plaque Agent, Miradent); and (3) light‐curing liquid (L; Plaque test, Ivoclar). The PDs were removed with dedicated toothbrushes (T_1_) and the specimens were repolished (T_2_). The protocol was repeated after 1 week of storage in artificial saliva (staining—T_3_ and repolishing—T_4_). Color measurement were performed at baseline (T_0_) and all testing times (T_1_—T_4_) using a recently introduced digital colorimeter (SmartColor, Smart Vision). Color changes (Δ*E*_ab_) compared to T_0_ were automatically recorded by the digital instrument. The data were statistically analyzed (*p* < 0.05).

**Results:**

The type of PD, the polishing procedure and their interactions influenced the color stability of both restorative materials (*p* < 0.05). Particularly, after the second PDs application, M and L produced the highest color changes (*p* < 0.05), with GIC showing higher color variability than RC. Although repolishing reduced the color changes of RC (*p* < 0.05), it could not reestablish the initial color of GIC, irrespective of the PD used (*p* < 0.05). Except for RC associated with T, all materials exhibited discoloration above the clinical perceptibility (1.77) and acceptability (2.66) thresholds.

**Conclusions:**

The influence of PDs on the color stability of RC and GIC was material‐dependent. GIC showed higher color instability than RC. Repolishing could not reestablish the original color of GIC and only attenuated the color changes of the one‐shade RC. The newly introduced digital colorimeter was an important tool to standardize and simplify color measurement evaluations.

**Clinical Significance:**

PDs can pose a potential risk to the color stability of restorative materials. Dental practitioners should be careful when recommending the frequency of at‐home application of PDs, taking into consideration the material properties and the position of the restorations of each patient.

## 1 Introduction

Dental plaque consists of several layers of bacterial colonies adhering to each other, forming a biofilm over the tooth surface [1]. During professional oral hygiene sessions, clinicians aim to remove supra‐ and subgingival tartar deposits, promote the patient's oral health, and recommend the most suitable techniques and aids for maintaining good oral hygiene at home. Although dental plaque is inherently colorless making it difficult to detect with bare eye [1, 2], its chemical and structural composition makes it prone to staining [1, 3]. Therefore, the rationale of the use of plaque detectors is to assist clinicians during professional oral hygiene procedures and to encourage patients to improve motivation at home oral hygiene [4]. There are currently no clear guidelines on the recommended frequency of use for these agents. Their application is typically advised based on each patient's caries risk level. For patients with a high risk of caries or poor at‐home oral hygiene, frequent use of plaque detectors may be recommended, as these products are generally considered safe. However, the potential impact of frequent use of agents containing dye solutions on the color stability of restorative materials remains unclear and warrants further investigation.

Plaque detectors are available in different forms for both in‐office and at‐home use, including mouthwashes, toothpastes, and tablets. Regardless of the form, their mechanism of action is the same: the disclosing agent deposits on the dental surface and stains in the presence of plaque. This makes it possible to make patients aware of the presence/absence and location of bacterial plaque and to identify its maturity stage [3, 4]. The pigments in plaque detectors have different molecular sizes, which affect their diffusion in the biofilm and their duration [5]. Even though plaque disclosing agents have an affinity for bacteria, they can also deposit on soft tissues near the teeth (gums, tongue, etc.) and on existing restorative materials [5, 6, 7]. Notably, higher plaque accumulation is expected on restorations with rough surfaces, often due to improper polishing procedures [8, 9].

Among the different filling materials available, resin composites and glass‐ionomer cements (GICs) are widely used in both adult and pediatric patients to restore dental morphology and esthetics after loss of tooth structure, that is, due to caries lesions [10, 11]. Despite the medium‐to‐long‐term fluoride release advantages of GICs, they are often used for temporary restorations, lining materials, or definitive fillings in small lesions on non‐occlusal loaded areas. Improvements in the mechanical and esthetic properties make resin composite materials the first choice for the rehabilitation of both anterior and posterior teeth [12, 13, 14]. One‐shade resin composites are among the latest materials introduced, designed to simplify restorative procedures by limiting the number of shades required [15, 16, 17].

Although they have different compositions, the literature identifies discoloration as a common drawback of both resin composites and GICs when exposed to staining solutions [18, 19, 20]. Polishing of the restorations enhanced to mitigate material's discoloration in a product‐related manner [21, 22, 23, 24, 25, 26]. Whether the color variation is due to intrinsic factors (such as deficits in the material's inner structure) [27, 28, 29, 30, 31] or extrinsic factors (such as contact with dye solutions) [27, 30, 31, 32, 33, 34, 35], the esthetic result of the final restoration may be impaired [35, 36]. This could necessitate reintervention, potentially reducing the restoration's lifespan and causing patient dissatisfaction.

Several staining solutions have been used in literature to simulate oral conditions and test the color stability of restorative materials [36, 37, 38, 39, 40, 41, 42, 43] by means of specific tools (i.e., colorimeters and spectrophotometers) [44, 45, 46]. Previous studies have shown that plaque disclosure solutions can influence the color stability of different sealant materials [47] causing color changes beyond clinically acceptable thresholds [48]. However, to the best of the authors' knowledge, information on whether the use of plaque detectors could influence the color stability of resin composite or glass‐ionomer materials commonly used for restorative purposes is scarce [49].

Therefore, this laboratory study aimed to evaluate the effects of three types of plaque detectors on the color stability of a one‐shade resin composite and a GIC. Specifically, the null hypothesis tested was that the type of plaque detector has not influence on the color changes of the two materials tested.

## 2 Materials and Methods

### 2.1 Specimen Preparation

Two restorative materials were used in the study (*n* = 30): a light‐cured one‐shade nanocomposite resin (Venus Diamond One, Kulzer, Hanau, Germany) and a self‐cure GIC (Fuji IX GP Capsule A2, GC, Tokyo, Japan). A silicone rectangular mold (7 × 3 × 2 mm) was used for specimens preparation [50]. Materials were handled strictly following manufacturers' instructions and their complete formulations are listed in Table 1.

**TABLE 1:** Details of the materials used in the study.

Material	Manufacturer	Composition
Venus diamond one (LOT: K010200)	Kulzer, Hanau, Germany	Matrix: UDMA, TCD‐DI‐HEA, TEGDMA Filler: BaAlF, SiO_2_ (64 vol%; 81 wt%; 5 nm—20 μm in *ø*)
Fuji IX GP capsule (A2) (LOT: 210510A)	GC Corp., Tokyo, Japan	Powder: Fluoro‐alumino‐silicate glass, polyacrylic acid, pigment Liquid: Water, polyacrylic acid, carboxylic acid
Fuji varnish (LOT: 2102101)	GC Corp., Tokyo, Japan	50%–70% Isopropyl acetate, 20%–30% acetone
Plaq‐search (LOT: 5017042)	TePe, Malmö, Sweden	Hydrated dextrates, magnesium stearate, sodium starch glycolate, aroma, CI 42090, CI 45410
Plaque agent (LOT: 630126)	Miradent, Duisburg, Germany	Aqua, xylitol, glycerin, PEG‐40 hydrogenated castor oil, sodium benzoate, poloxamer 407, potassium sorbate, aroma, limonene, sodium chloride, sodium sulfate, citric acid, CI 42090
Plaque test indicator liquid (LOT: Z030FR)	Ivoclar, Schaan, Liechtenstein	Glycerin, aqua, CI 45350, potassium phosphate, ethylparaben, sodium hydroxide

Abbreviations: BaAlF = barium–aluminum–fluoride glass; CI 42090 = brilliant blue; CI 45350 = fluorescein, yellow; CI 45410 = phloxine, red dye; *ø* = diameter; SiO_2_ = silica; TCD‐DI‐HEA = 2‐propenoic acid (octahydro‐4,7‐methano‐1*H*‐indene‐5‐diyl) bis(methyleneiminocarbonyloxy‐2,1‐ethanediyl) ester; TEGDMA = triethylene glycol dimethacrylate; UDMA = urethane dimethacrylate.

The RC was compacted in two 1‐mm‐thick layers into the silicone mold. Then, a Mylar strip was placed on the top of each resin specimen, and light‐polymerization was performed with a light‐emitting diode lamp for 20 s (LED; SmartLite Pro, Dentsply Sirona, Konstanz, Germany; wavelength: 450–480 nm; light output: 1550–600 mW/cm^2^). An additional light‐curing for 40 s was performed after the removal of the transparent strip on the top of the specimen and on the bottom surface previously in contact with the mold.

The GIC was centrifugated for 10 s and extruded in one single mass within 10 s with a disposable syringe filling the silicone mold. A transparent Mylar strip was applied on the top of the specimen to make the surface smoother until the complete setting of the material (approximately 2 min and 30 s). When set, a protective coating (Fujy Varnish, GC) was applied with a microbrush over the entire specimen's surface, as suggested by manufacturer. The varnish was gently air‐dried and maintained in place for 2 min.

After the preparation, the specimens were wet polished with increasing SiC papers grain size (1.200 and 4.000 grain size, respectively) and then stored in artificial saliva (KCl [12.92 mmol/L], KSCN [1.95 mmol/L], Na_2_SO_4_·10H_2_O [2.37 mmol/L], NH_4_Cl [3.33 mmol/L], CaCl_2_·2H_2_O [1.55 mmol/L], NaHCO_3_ [7.51 mmol/L], and ZnCl_2_ [0.02 mmol/L] in HEPES buffer solution) [47] in a laboratory oven at 37°C to allow for the complete setting of the materials.

### 2.2 Plaque Detector Application

Three plaque detectors with different formulations (Table 1) and delivery forms were used: (1) tablets (Plaq‐Search, TePe, Malmö, Sweden); (2) mouthwash (Plaque Agent, Miradent, Duisburg, Germany); and (3) light‐curing disclosure liquid (Plaque Test Indicator Liquid, Ivoclar, Schaan, Liechtenstein). One soft bristle toothbrush (Special Care, Tepe Oral Health Care, Anaheim, CA, USA) was singularly used per each group to remove the plaque agents, as per manufacturer's instructions. The saliva of a healthy operator who abstained from eating and drinking in the previous 2 h was collected in a sterile Eppendorf container and used for PDs' preparation [47]. Briefly, the following procedures were used:

1. Tablets (T): A tablet of detector liquid was dissolved into the Eppendorf containing the saliva sample. Then, a cotton swab was used to apply the solution on the samples for 10 s. Afterward, each specimen was rinsed under running water and brushed for 2 min with its dedicated toothbrush as to reproduce clinical conditions to remove excesses of the disclosure agent and then the specimen was air‐dried.
2. Mouthwash (M): 0.25 mL of mouthwash was mixed with 0.5 mL of saliva. The solution was mixed and applied to each sample with a cotton swab for 30 s. At the end, the specimens were brushed with a dedicated brush under running water for 2 min, as previously described, and air‐dried.
3. Light‐curing liquid (L): One drop of saliva was swab with a cotton pellet over each specimen. Then, one drop of liquid was applied with another cotton swab for 3 s and the specimen rinsed with water and gently air‐dried. By the activation of the curing lamp (SmartLite Pro, Dentsply Sirona, Charlotte, NC, USA), the amount of plaque was highlighted as fluorescent yellow. After 2 min, the specimens were brushed with a soft toothbrush for 2 min under running water and air‐dried.

The plaque detectors were applied twice, 1 week apart, to simulate normal domiciliary use [16]. During the application interval, the specimens were maintained in artificial saliva.

### 2.3 Color Measurements

**FIGURE 1:**
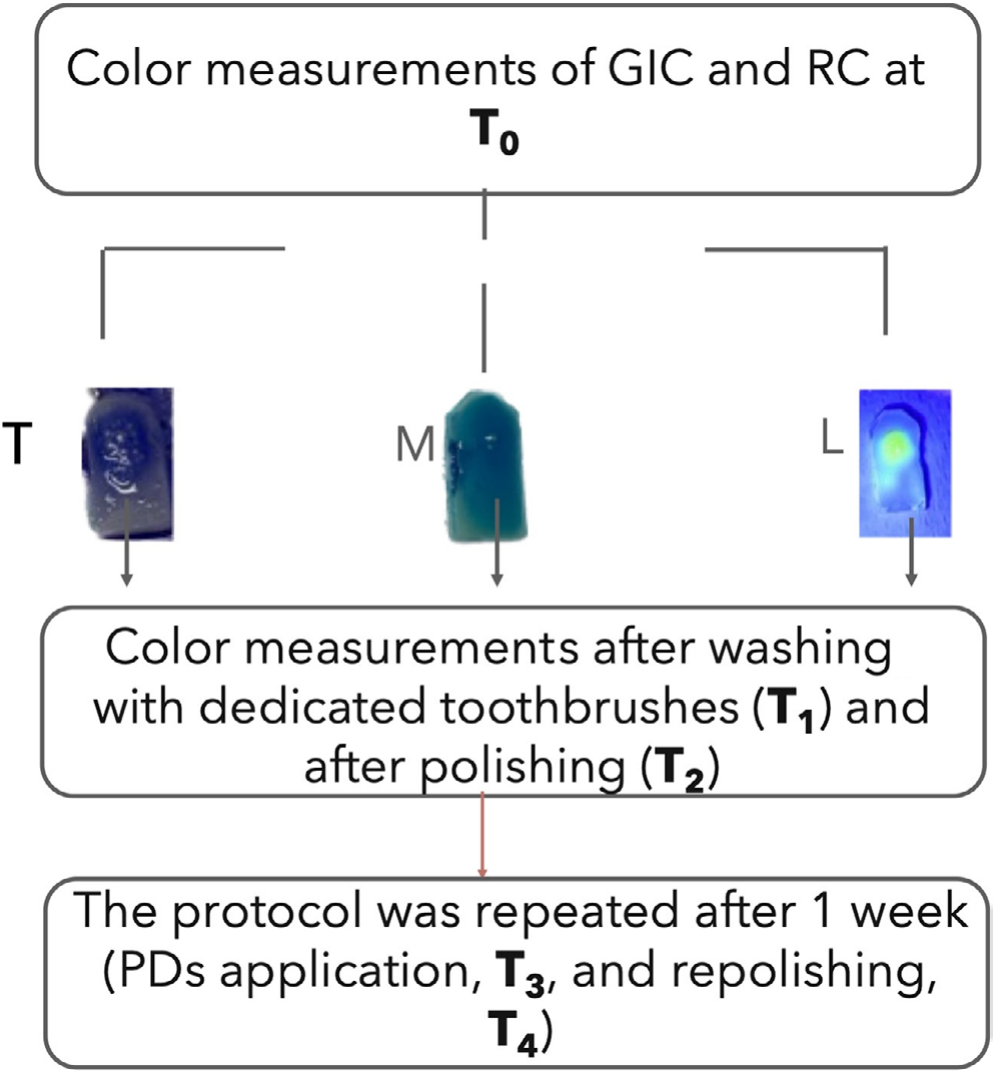
The diagram schematizes the timepoints and experimental procedures used in the study. GIC: glass ionomer cement; L: liquid; M: mouthwash; RC: resin composite; T: tablets.

The color coordinates, L* (brightness), a* (red/green values), and b* (blue/yellow values), of the CIELab model system were obtained at different stages using a latest generation of a live‐video comparative spot colorimeter (Easy_Color, SmartVision, Udine, Italy). CIE Lab settings were as follows: observer degree: 2; ∆*E* type: ∆*E*_ab_ CIELAB; illuminant: *D*; temperature: 50. White and black calibration was performed as suggested by the manufacturer at the beginning of the test and after each group was completed. Once the software alerts the calibration has been passed, each specimen was evaluated immediately after 24 h from preparation and polishing (T_0_), after the first round of staining (T_1_), after the first repolishing (T_2_), after the second round of staining (T_3_), and after the second repolishing procedures (T_4_). First and second repolishing (T_2_ and T_4_, respectively) followed the same procedures performed at T_0_. The protocol flowchart is summarized in Figure 1. The first measurement (T_0_) was used to record the master model, which serves as the reference for evaluating any color deviations in subsequent measurements of specimens from the same group.

Measurements were performed by one trained operator. Briefly, each specimen was positioned over the reading glass provided with an LED light source and covered with a black cap. The position and size of the analysis area (1.73 × 0.86 mm) were adjusted and customized. This system allowed for repeated measurements at the same location and with the same measurement size on the same specimens for repeated measurements, or in different specimens. This enabled standardization and repeatability of measurements at the same site across different test stages. The instrument allowed for accurate one‐shot color analysis (2 s of duration). The color of a reference master model was taken (T_0_) per each specimen. Then, the reference L*a*b* values were compared to the current ones, and the Δ*E* values were automatically recorded by the system. The thresholds were set at ∆*E*_ab_ = 1.22 and ∆*E*_ab_ = 2.66 (according to the 50%:50% perceptibility, PT, and 50%:50% acceptability, AT, respectively) [48].

After checking the normal and equal distribution (Shapiro–Wilk and Brown–Forsythe tests (*p* > 0.05), respectively), a two‐way analysis of variance (ANOVA) and All Pairwise Multiple Comparison Procedures (Holm–Sidak method) was run separately for each restorative material, with the type of plaque detector and different time points as independent variables, and the color values as the dependent variable. All tests were performed using SigmaPlot v. 14.0 (Systat Software GmbH, Düsseldorf, Germany). The significance level was set at *p* < 0.05.

## 3 Results

**FIGURE 2:**
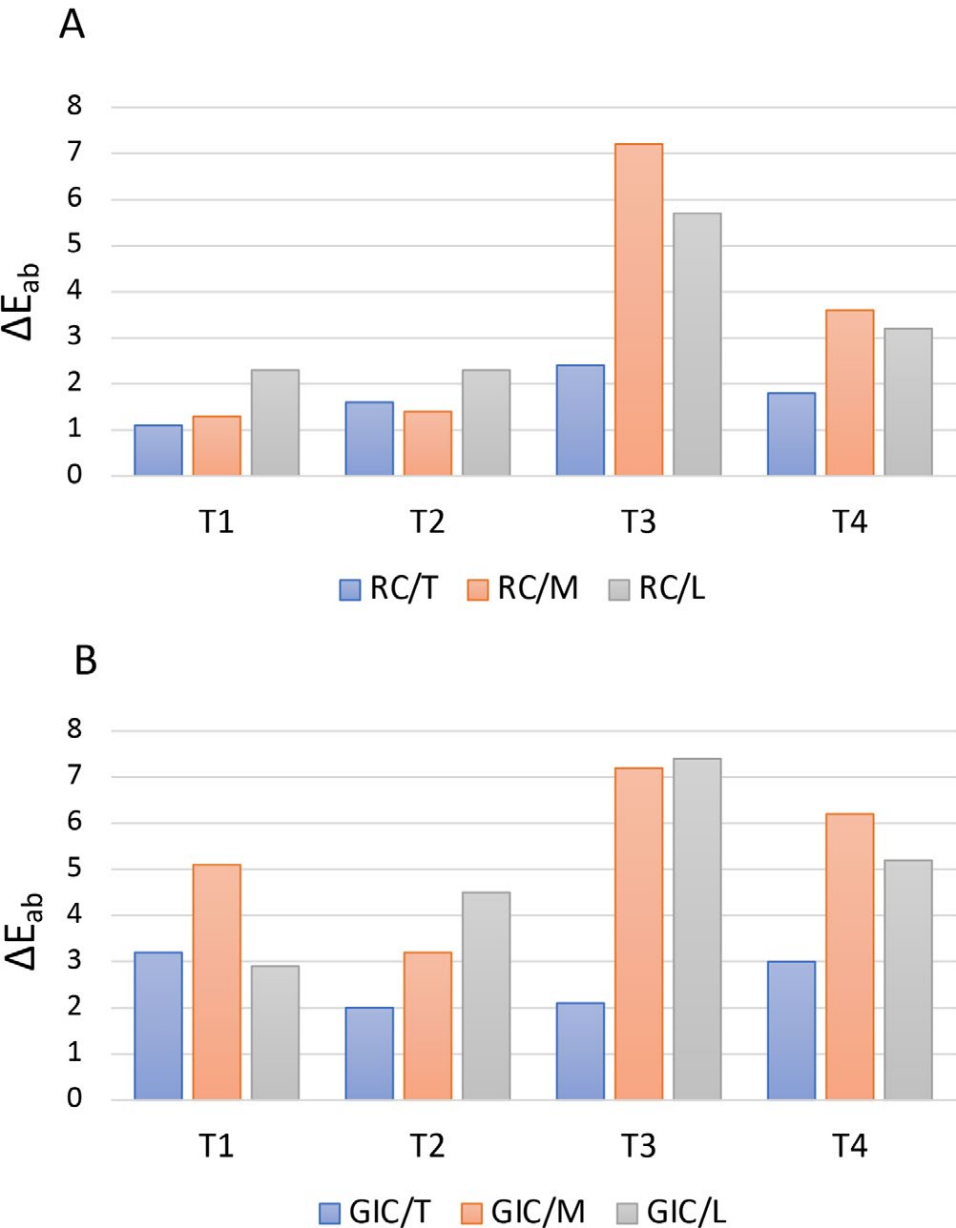
Bar charts representing the color differences (Δ*E*) among groups RC (A) and GIC (B) at the different testing timepoints after application of plaque detectors. GIC: glass ionomer cement; L: liquid; M: mouthwash; RC: resin composite; T: tablets.

The results of the color differences (∆*E*_ab_) before and after using the different plaque detector agents with the two restorative materials are reported in Table 2A,B and Figure 2A,B.

**TABLE 2:** Means (standard deviations) of the Δ*E*_ab_ in the resin composite (A) and the glass ionomer cement (B) groups. After the second application, all materials overpassed the acceptability and perceptibility thresholds, irrespective of the plaque detectors used, apart RC/T that was the only group relying within the acceptable clinical parameter. Even though repolishing could reduce the color changes in the RC groups (A), the same procedure did not show the same effectiveness in the GIC groups (B).

Materials	T1	T2	T3	T4
(A) RC				
Tablet (T)	1.1 (0.5)^A1^	1.6 (0.5)^AB1^	2.4 (1)^B1^	1.8 (0.6)^AB1^
Mouthwash (M)	1.3 (0.4)^A2^	1.4 (0.3)^A1^	7.2 (0.3)^C3^	3.6 (0.7)^B2^
Liquid (L)	2.3 (0.7)^A3^	2.3 (0.8)^A2^	5.7 (1.3)^C2^	3.2 (0.6)^B2^
(B) GIC				
Tablet (T)	3.2 (1.5)^A1^	2 (1.6)^A1^	2.1 (1.7)^A1^	3 (2)^A1^
Mouthwash (M)	5.1 (2.2)^AB1^	3.2 (1.5)^A1,2^	7.2 (1.6)^B2^	6.2 (1.2)^B2^
Liquid (L)	2.9 (1.9)^A1^	4.5 (1.8)^A2^	7.4 (3.4)^B2^	5.2 (2.3)^B2^

*Note*: Letter in each row and numbers in each column indicate statistically significant differences (*p* < 0.05).

The type of plaque detector, the polishing procedures and the interaction between these factors significantly influenced the color stability of both RC and GIC (*p* < 0.001). In particular, the second application of the plaque detectors was found to be more influential on the discoloration of the tested materials (*p* < 0.05).

Apart from the RC/T which was the only group that fell below the range of acceptable clinical perceptibility, the other groups were all above the PT and AT thresholds, particularly, evident for the GIC. Among the plaque detectors, M and L resulted in higher color changes compared to T (*p* < 0.05). Although the effectiveness of the polishing was material/product‐dependent, after the second application of the plaque detectors, it was not possible to recover the initial color. These changes were more visible after the use of M and L.

Within the resin composite group, the PDs differently influenced the color stability (*p* < 0.05). After the first application, T and M stained less, followed by L. After the second PD application, M and L obtained the highest color changes among the groups. Repolishing after the first application (T_2_) was able to restore the initial color of the RC. However, after the second application, M showed the highest color changes, followed by L and T (*p* < 0.05). In this case, the second repolishing (T_4_) statistically reduced the color changes but did not reestablish the initial appearance.

When analyzing the effects of PDs on the color stability of the GIC group, it was demonstrated that L and M yielded the highest discoloration (*p* < 0.05). In these groups, repolishing reduced the differences, but it could not recover the initial color of the material (*p* < 0.05). The second application of all PDs negatively influenced the color stability of the GIC, particularly evident for M and L (*p* < 0.05) and, in this case, repolishing was statistically ineffective in restoring the initial color of the material (*p* < 0.05).

## 4 Discussion

In this study, plaque detectors affected the color stability of the restorative materials tested and the results were different according to their delivery forms. Therefore, the null hypothesis must be rejected.

The increasing use of plaque detectors in both clinical settings and at home is primarily driven by their ability to stain dental plaque, making it easier for patients and clinicians to disclose and manage oral hygiene maneuvers, aiding in patient education and motivation [6]. However, the staining capability of PDs has raised concerns about their potential impact on the color stability of restorative materials [47, 49]. The literature has extensively documented the staining effects of various substances, including oral hygiene aids such as mouthwashes or toothpastes on restorative materials [39, 40, 41, 42]. In an era of high patients' esthetic demand and increasingly advanced materials and technologies, color variation could create economic and satisfaction problems.

Plaque detectors differ in terms of composition and delivery forms. The latter are aimed to gain both pediatric and adult patients' collaboration. Regarding the composition, they may contain different aromas to make their use more pleasant combined with one (monotone) or more (bi‐ or tri‐tone) colorants such as CI 42090 (brilliant blue), CI 45410 (phloxine, red dye), CI 45430 (erythrosine), CI 45350 (fluorescein), CI 42053 (fast green), CI 42051 (patent bleu), and CI 16035 (allura red) that define their hue. This characteristic allows to differentiate the new and old plaque by exhibiting different staining of the dental surface [1, 3, 6], as demonstrated by the tablets (bi‐tone with brilliant blue and red dyes) and mouthwash (mono‐tone based on brilliant blue dye) products used in this study. These dyes are not new in the field of biotechnology, as they are often used for the study of biological tissues processed for microscopy evaluations. This is because of the presence of acidic groups in these colorants that chemically interact with the substrates they come in contact with [7].

Unlike the tablets and the mouthwash, the liquid plaque detector used in this study is based on sodium salts of fluorescein (Table 1). This solution is applied on the surface of the teeth before the professional hygiene session and activated by a blue LED lamp to expose the plaque on the teeth, making it fluorescent yellow. The results of this study indicated that the liquid, together with the mouthwash, caused greater discoloration than tablets. This phenomenon can be hypothetically attributed to the presence of dye residues on the materials' surfaces even after washing, and this contamination can significantly impact the materials' discoloration and properties [7]. These results are not in accordance with those achieved by Maki Hino et al., who found little influence of the fluorescent dye on the tooth‐colored materials tested [20]. Apart from the different restorative materials tested, it should be noted that a different protocol was applied in the study of Maki Hino et al., where the disclosing agents were applied only once and no polishing was performed, which was reported by the same authors as limitation of their study [49]. On the other hand, a limitation of our protocol is that the results of the liquid PD can hardly be extrapolated into a clinical context, since, unlike the other two products, the liquid is a device for professional use only, so it is assumed that it is unrealistic that the application of the product will be repeated at a distance of a week only, reducing the possibility that the dye could influence the final esthetic result of the restorative material in this form.

The esthetic success of resin‐based composite restorations depends, to a large extent, on their optical properties which are supposed to mimic those of natural teeth [10, 11, 48]. For a natural appearance, resin composites should have a visual shading and translucency similar to the corresponding tooth properties [12]. Recently, “one‐shade” composites have appeared on the market, characterized by their ability to acquire a single universal shade, therefore adaptable to differently colored substrates [16].

Except when liquid was used, the first application of the plaque detector did not result in significant color changes of the composite resin used in the study after polishing (Table 2A and Figure 2A). However, after 1 week of storage in artificial saliva and a second application of the plaque detector, a notable decline in color stability was observed, particularly evident with the mouthwash and the liquid disclosure agent. Although polishing reduced the extent of staining, it could not completely restore the original color. This suggests that repeated exposure to plaque detectors, combined with the natural aging process in the oral environment, can compromise the esthetic longevity of composite restorations. The filler composition (including particle size and distribution) [32], the matrix as well as the efficacy of polymerization [27] are not only relevant to characterize mechanical properties of resin‐based materials but play also a crucial role in the material's susceptibility to staining and, eventually, its response to polishing [33]. In particular, the one shade composite tested possesses irregularly shaped fillers along with smaller submicron particles dispersed in a resinous matrix formed of UDMA, TCD‐DI‐HEA, and TEGDMA which confer the material an optimized polymer cross‐linking that enhances the polymerization process [28, 29]. However, acqueous solutions, such as saliva [27, 34], are attracted by hydrophilic low molecular weight (i.e., TEGDMA) and highly tough (i.e., UDMA) monomers [27], particularly, when not properly converted [39]. In this case, saliva can diffuse into the polymeric network determining swelling phenomena and creating micropores [30], possibly causing the entrapment of traces of the dyes of the PDs difficult to be removed with repolishing [36].

According to our results, the discoloration of the GIC progressively increased after the second application, irrespective of the plaque detector used (Table 2B and Figure 2B). This finding can be attributed to the inherent properties of GICs, which include higher porosity, greater water sorption, and solubility compared to resin‐based materials [20]. It should also be noted that not only colorants but also flavorings and other mainly acidic components added to the staining media (Table 1) can cause structural changes such as softening of the materials' structure, internal cracking and increased surface roughness [31].

The effectiveness of repolishing in mitigating the staining effects of plaque detectors was evident [21], but our results indicated that its ability varied between the two materials tested. The type of polishing instrument (e.g., burs, discs, brushes) and the polishing protocol (e.g., duration, pressure applied) can significantly affect the surface smoothness and, consequently, the material's resistance to staining [22, 23, 24]. In this study, while repolishing improved the color stability of RC after the first plaque detector application, its effectiveness diminished after the second application and subsequent storage. In the case of GIC, repolishing was ineffective in restoring the original color nor moderating the discoloration. Concerns have been previously raised on the smoothness and surface texture quality of GICs after polishing procedures [25, 26]. Surface roughness translates into the increased likelihood of external colorants incorporation [18], leading to higher discoloration upon the second application of the plaque detectors, as observed in this study. This underscores the need for optimized polishing techniques tailored to the specific properties of each restorative material [18], in particular, when esthetic restoration needs to be continuously refined [40].

Due to the unique oral environment, which includes exposure to various foods, beverages, and other factors that can affect color [38], along with the often‐complex composition of dental materials, it appears that no material can be considered completely color‐stable to date. Studies investigating the color stability of dental materials have primarily utilized instruments such as colorimeters and spectrophotometers [44, 45]. However, there is a common consensus that these tests are time‐consuming and susceptible to various influences, including external light, sample conditions, operator variability etc. [46] Advancements in digital technology offer a promising solution to mitigate these variables and achieve more accurate and consistent results. In this study, a latest‐generation digital colorimeter, traditionally used for quality control analyses and very recently adapted to dental purposes, was employed for the color evaluations. The instrument consists in a live‐video comparative spot colorimeter in diffusive light reflection able to show the real‐time image of specimens to be measured. The standardized features of this instrument, the digitalization of measurements and the possibility to customize the parameters (i.e., analysis slot and location, *ΔE calculation method*) according to the study design not only allowed for significantly faster testing but also minimized external influences from light and operator handling and evaluation, thereby enhancing the reproducibility of the results over time.

The findings of this study have relevant implications for clinical practice. Given the propensity of plaque detectors to stain restorative materials, clinicians should carefully consider the frequency and type of plaque detectors used, especially in patients with extensive and esthetically impactful restorations. Additionally, the choice of restorative material should consider not only the mechanical and esthetic properties but also the material's resistance to staining and ease of polishing. For GICs, which are often used in pediatric dentistry and as temporary restorations, the higher susceptibility to staining highlights the need for improved formulations with enhanced resistance to discoloration. For composite resins, ongoing research on filler technology and surface treatments could further improve their color stability and resistance to staining.

Future studies should explore the long‐term effects of plaque detectors on a wider range of restorative materials and under more varied conditions that simulate the oral environment, including the effect of saliva on materials' resistance to discoloration. Indeed, this study did not include a group immersed solely in saliva, which could be considered a limitation. However, it is important to note that previous research has shown no color changes in samples immersed in artificial saliva for up to 14 days, with slight discoloration observed only after 21 days of immersion [35]. It has also been reported that composite filler leaching is significantly higher in artificial saliva compared to distilled water [43]. Saliva and the subsequent accumulation of a pellicle layer act as a matrix for the deposition of stains, which may contribute to discoloration [40].

The absence of a saliva‐only group in our study may limit our understanding of these effects, particularly considering that filler leaching can increase surface roughness, thereby enhancing the potential for staining [40]. Nonetheless, these effects have been reported to become evident only after prolonged exposure, such as 21 days of testing [35]. Considering the 2‐week duration of our study, we cautiously hypothesize that this time frame was insufficient to induce noticeable color changes attributable to saliva. However, these hypotheses should be further investigated in future studies.

Additionally, investigating different polishing tools and protocols could provide deeper insights into optimizing restorative maintenance. Another area of interest is the impact of staining on the blending effect of one‐shade composites, which are designed to match a wide range of tooth shades [17]. Any compromise in color stability could undermine the esthetic benefits of these materials.

In conclusion, despite their important role in promoting oral hygiene, plaque detectors seem to pose a potential risk to the color stability of restorative materials. Dental practitioners should be careful when recommending the frequency of at‐home application of plaque detectors, taking into consideration the material properties and the position of each patient's restorations. Careful polishing procedures should be performed during each visit to limit material discoloration and modify surface texture.

## 5 Conclusions

Within the limitation of this study, the use of different types of plaque detectors resulted in a color change for both the GIC and one‐shade resin composite tested, exceeding the threshold of clinical acceptability. Specifically, the frequency of applications and the effectiveness of polishing were critical factors in the outcomes observed. However, further studies are necessary to explore these findings in greater detail.

## Author Contributions

**Claudia Mazzitelli:** conceptualization, methodology, writing – original draft preparation, supervision. **Gaetano Paolone:** conceptualization, methodology, writing – original draft preparation. **Uros Josic:** formal analysis and investigation. **Edoardo Mancuso:** formal analysis and investigation. **Alessandro Vichi:** writing – original draft preparation, writing – review and editing. **Ginevra Pastremoli:** formal analysis and investigation. **Annalisa Mazzoni:** writing – review and editing. **Lorenzo Breschi:** writing – review and editing, supervision. **Tatjana Maravic:** methodology: writing – original draft preparation.

## Conflicts of Interest

The authors declare no conflicts of interest.

## Data Availability

The data that support the findings of this study are available from the corresponding author upon reasonable request.
